# Supplementary material for: Modeling glioblastoma heterogeneity as a dynamic network of cell states
Source: Mol Syst Biol. 2021 Sep 16;17(9):e10105. doi: 10.15252/msb.202010105 (PMC8444284; doi:10.15252/msb.202010105)
Supplement: Supplementary file 6 — Source Data for Figure 5 [file MSB-17-e10105-s004.zip › Figure5A_sourcedata/GSEA_3017/nosigdb_stateB.GseaPreranked.1622795021725/RICHARDS_NOWAKOWSKI_SCIENCE_2017_IPC_DIV1_UPREG.html]

Details for gene set RICHARDS\_NOWAKOWSKI\_SCIENCE\_2017\_IPC\_DIV1\_UPREG[GSEA]

|  || Dataset | state43017 |
| Phenotype | NoPhenotypeAvailable |
| Upregulated in class | na\_pos |
| GeneSet | RICHARDS\_NOWAKOWSKI\_SCIENCE\_2017\_IPC\_DIV1\_UPREG |
| Enrichment Score (ES) | 0.51751626 |
| Normalized Enrichment Score (NES) | 3.331615 |
| Nominal p-value | 0.0 |
| FDR q-value | 0.0 |
| FWER p-Value | 0.0 |
Table: GSEA Results Summary

  

Fig 1: Enrichment plot: RICHARDS\_NOWAKOWSKI\_SCIENCE\_2017\_IPC\_DIV1\_UPREG      
 Profile of the Running ES Score & Positions of GeneSet Members on the Rank Ordered List

  

| PROBE | GENE SYMBOL | GENE\_TITLE | RANK IN GENE LIST | RANK METRIC SCORE | RUNNING ES | CORE ENRICHMENT || 1 | HIST1H4C |  |  | 0 | 1.373 | 0.0109 | Yes |
| 2 | HIST1H1B |  |  | 1 | 1.272 | 0.0211 | Yes |
| 3 | HIST1H1D |  |  | 2 | 1.180 | 0.0305 | Yes |
| 4 | TOP2A |  |  | 3 | 1.079 | 0.0391 | Yes |
| 5 | HIST1H1C |  |  | 4 | 1.041 | 0.0473 | Yes |
| 6 | RRM2 |  |  | 7 | 0.875 | 0.0502 | Yes |
| 7 | FAM111A |  |  | 10 | 0.813 | 0.0525 | Yes |
| 8 | UBE2C |  |  | 11 | 0.802 | 0.0589 | Yes |
| 9 | TPX2 |  |  | 12 | 0.801 | 0.0653 | Yes |
| 10 | NUSAP1 |  |  | 13 | 0.801 | 0.0717 | Yes |
| 11 | HMGB2 |  |  | 14 | 0.786 | 0.0779 | Yes |
| 12 | FBXO5 |  |  | 15 | 0.785 | 0.0842 | Yes |
| 13 | NCAPG |  |  | 16 | 0.778 | 0.0904 | Yes |
| 14 | SPC25 |  |  | 17 | 0.763 | 0.0964 | Yes |
| 15 | PRC1 |  |  | 18 | 0.756 | 0.1025 | Yes |
| 16 | CDCA2 |  |  | 19 | 0.751 | 0.1084 | Yes |
| 17 | ESCO2 |  |  | 20 | 0.742 | 0.1144 | Yes |
| 18 | TACC3 |  |  | 21 | 0.741 | 0.1203 | Yes |
| 19 | GTSE1 |  |  | 22 | 0.732 | 0.1261 | Yes |
| 20 | HJURP |  |  | 23 | 0.731 | 0.1319 | Yes |
| 21 | KIF23 |  |  | 24 | 0.721 | 0.1376 | Yes |
| 22 | ASPM |  |  | 26 | 0.695 | 0.1411 | Yes |
| 23 | NDC80 |  |  | 27 | 0.695 | 0.1466 | Yes |
| 24 | ATAD2 |  |  | 28 | 0.685 | 0.1521 | Yes |
| 25 | KIF15 |  |  | 29 | 0.684 | 0.1575 | Yes |
| 26 | ARHGAP11A |  |  | 30 | 0.683 | 0.1630 | Yes |
| 27 | KPNA2 |  |  | 33 | 0.676 | 0.1642 | Yes |
| 28 | CENPF |  |  | 34 | 0.670 | 0.1696 | Yes |
| 29 | KIF4A |  |  | 37 | 0.662 | 0.1707 | Yes |
| 30 | ANLN |  |  | 39 | 0.659 | 0.1739 | Yes |
| 31 | RAD51AP1 |  |  | 40 | 0.657 | 0.1791 | Yes |
| 32 | SMC4 |  |  | 41 | 0.655 | 0.1843 | Yes |
| 33 | MELK |  |  | 42 | 0.655 | 0.1895 | Yes |
| 34 | CENPE |  |  | 43 | 0.654 | 0.1947 | Yes |
| 35 | NUF2 |  |  | 44 | 0.651 | 0.1999 | Yes |
| 36 | PRR11 |  |  | 45 | 0.649 | 0.2051 | Yes |
| 37 | DLGAP5 |  |  | 46 | 0.647 | 0.2103 | Yes |
| 38 | KIFC1 |  |  | 47 | 0.645 | 0.2154 | Yes |
| 39 | KIF18A |  |  | 48 | 0.639 | 0.2205 | Yes |
| 40 | NCAPG2 |  |  | 50 | 0.636 | 0.2235 | Yes |
| 41 | CENPA |  |  | 51 | 0.636 | 0.2285 | Yes |
| 42 | CDCA5 |  |  | 52 | 0.632 | 0.2336 | Yes |
| 43 | CKAP2 |  |  | 53 | 0.628 | 0.2386 | Yes |
| 44 | CCNA2 |  |  | 54 | 0.626 | 0.2436 | Yes |
| 45 | AURKA |  |  | 55 | 0.617 | 0.2485 | Yes |
| 46 | FANCI |  |  | 56 | 0.615 | 0.2534 | Yes |
| 47 | CKAP2L |  |  | 59 | 0.611 | 0.2541 | Yes |
| 48 | CENPU |  |  | 60 | 0.605 | 0.2589 | Yes |
| 49 | TAGLN2 |  |  | 61 | 0.604 | 0.2637 | Yes |
| 50 | CENPK |  |  | 62 | 0.603 | 0.2685 | Yes |
| 51 | TYMS |  |  | 63 | 0.601 | 0.2733 | Yes |
| 52 | BUB1B |  |  | 64 | 0.596 | 0.2781 | Yes |
| 53 | CCDC34 |  |  | 65 | 0.595 | 0.2828 | Yes |
| 54 | KIF2C |  |  | 66 | 0.591 | 0.2875 | Yes |
| 55 | SMC2 |  |  | 68 | 0.586 | 0.2901 | Yes |
| 56 | CDK1 |  |  | 69 | 0.585 | 0.2948 | Yes |
| 57 | H2AFX |  |  | 71 | 0.576 | 0.2973 | Yes |
| 58 | CKS1B |  |  | 72 | 0.575 | 0.3018 | Yes |
| 59 | RACGAP1 |  |  | 75 | 0.572 | 0.3023 | Yes |
| 60 | FOXM1 |  |  | 76 | 0.570 | 0.3068 | Yes |
| 61 | MAD2L1 |  |  | 77 | 0.569 | 0.3113 | Yes |
| 62 | PLK1 |  |  | 78 | 0.567 | 0.3158 | Yes |
| 63 | DBF4 |  |  | 79 | 0.567 | 0.3203 | Yes |
| 64 | CDCA8 |  |  | 82 | 0.562 | 0.3207 | Yes |
| 65 | CKS2 |  |  | 83 | 0.558 | 0.3251 | Yes |
| 66 | ECT2 |  |  | 84 | 0.558 | 0.3296 | Yes |
| 67 | KIF14 |  |  | 85 | 0.557 | 0.3340 | Yes |
| 68 | E2F7 |  |  | 86 | 0.556 | 0.3384 | Yes |
| 69 | AURKB |  |  | 89 | 0.548 | 0.3387 | Yes |
| 70 | PIF1 |  |  | 90 | 0.545 | 0.3430 | Yes |
| 71 | SPC24 |  |  | 91 | 0.544 | 0.3473 | Yes |
| 72 | CENPM |  |  | 92 | 0.544 | 0.3517 | Yes |
| 73 | CCNB1 |  |  | 93 | 0.544 | 0.3560 | Yes |
| 74 | NCAPH |  |  | 94 | 0.543 | 0.3603 | Yes |
| 75 | PBK |  |  | 95 | 0.540 | 0.3646 | Yes |
| 76 | CSRP2 |  |  | 96 | 0.539 | 0.3689 | Yes |
| 77 | DEPDC1 |  |  | 97 | 0.536 | 0.3732 | Yes |
| 78 | XRCC2 |  |  | 98 | 0.530 | 0.3774 | Yes |
| 79 | SKA3 |  |  | 99 | 0.525 | 0.3816 | Yes |
| 80 | RRM1 |  |  | 103 | 0.520 | 0.3795 | Yes |
| 81 | CMC2 |  |  | 105 | 0.516 | 0.3816 | Yes |
| 82 | CCNF |  |  | 106 | 0.513 | 0.3856 | Yes |
| 83 | CDKN3 |  |  | 108 | 0.513 | 0.3877 | Yes |
| 84 | CEP135 |  |  | 109 | 0.513 | 0.3917 | Yes |
| 85 | UBE2T |  |  | 110 | 0.512 | 0.3958 | Yes |
| 86 | CDCA3 |  |  | 111 | 0.511 | 0.3999 | Yes |
| 87 | DIAPH3 |  |  | 112 | 0.510 | 0.4039 | Yes |
| 88 | BUB1 |  |  | 113 | 0.508 | 0.4080 | Yes |
| 89 | TUBB4B |  |  | 114 | 0.507 | 0.4120 | Yes |
| 90 | RAD21 |  |  | 115 | 0.506 | 0.4161 | Yes |
| 91 | BIRC5 |  |  | 116 | 0.504 | 0.4201 | Yes |
| 92 | CLSPN |  |  | 117 | 0.503 | 0.4241 | Yes |
| 93 | DTYMK |  |  | 118 | 0.503 | 0.4281 | Yes |
| 94 | CEP152 |  |  | 119 | 0.502 | 0.4321 | Yes |
| 95 | KIF18B |  |  | 123 | 0.494 | 0.4298 | Yes |
| 96 | SPDL1 |  |  | 126 | 0.492 | 0.4296 | Yes |
| 97 | TMPO |  |  | 127 | 0.492 | 0.4335 | Yes |
| 98 | ITGB3BP |  |  | 128 | 0.490 | 0.4374 | Yes |
| 99 | LMNB2 |  |  | 129 | 0.488 | 0.4413 | Yes |
| 100 | SKA1 |  |  | 131 | 0.487 | 0.4431 | Yes |
| 101 | SHCBP1 |  |  | 133 | 0.484 | 0.4449 | Yes |
| 102 | GMNN |  |  | 135 | 0.484 | 0.4467 | Yes |
| 103 | NCAPD2 |  |  | 136 | 0.483 | 0.4505 | Yes |
| 104 | TTK |  |  | 137 | 0.482 | 0.4544 | Yes |
| 105 | FANCB |  |  | 138 | 0.478 | 0.4582 | Yes |
| 106 | CENPL |  |  | 139 | 0.478 | 0.4620 | Yes |
| 107 | INCENP |  |  | 140 | 0.476 | 0.4658 | Yes |
| 108 | C21orf58 |  |  | 142 | 0.470 | 0.4674 | Yes |
| 109 | FANCD2 |  |  | 149 | 0.461 | 0.4587 | Yes |
| 110 | ORC6 |  |  | 150 | 0.461 | 0.4624 | Yes |
| 111 | KIF22 |  |  | 152 | 0.459 | 0.4639 | Yes |
| 112 | NCAPD3 |  |  | 153 | 0.458 | 0.4676 | Yes |
| 113 | KNSTRN |  |  | 155 | 0.456 | 0.4691 | Yes |
| 114 | SPAG5 |  |  | 156 | 0.455 | 0.4728 | Yes |
| 115 | MCM8 |  |  | 158 | 0.454 | 0.4743 | Yes |
| 116 | GPC6 |  |  | 159 | 0.454 | 0.4779 | Yes |
| 117 | CEP78 |  |  | 161 | 0.451 | 0.4794 | Yes |
| 118 | POLQ |  |  | 162 | 0.450 | 0.4830 | Yes |
| 119 | CENPQ |  |  | 163 | 0.446 | 0.4866 | Yes |
| 120 | TK1 |  |  | 167 | 0.444 | 0.4839 | Yes |
| 121 | BRIP1 |  |  | 168 | 0.443 | 0.4874 | Yes |
| 122 | ATAD5 |  |  | 170 | 0.442 | 0.4889 | Yes |
| 123 | BRCA1 |  |  | 171 | 0.440 | 0.4924 | Yes |
| 124 | CDK5RAP2 |  |  | 173 | 0.438 | 0.4938 | Yes |
| 125 | BRCA2 |  |  | 174 | 0.436 | 0.4973 | Yes |
| 126 | DEPDC1B |  |  | 177 | 0.436 | 0.4966 | Yes |
| 127 | MKI67 |  |  | 179 | 0.436 | 0.4980 | Yes |
| 128 | RHNO1 |  |  | 183 | 0.432 | 0.4952 | Yes |
| 129 | CDC20 |  |  | 184 | 0.431 | 0.4987 | Yes |
| 130 | USP1 |  |  | 185 | 0.430 | 0.5021 | Yes |
| 131 | MIS18BP1 |  |  | 186 | 0.430 | 0.5055 | Yes |
| 132 | BARD1 |  |  | 189 | 0.427 | 0.5048 | Yes |
| 133 | HMMR |  |  | 191 | 0.426 | 0.5061 | Yes |
| 134 | CKAP5 |  |  | 192 | 0.426 | 0.5095 | Yes |
| 135 | MND1 |  |  | 193 | 0.424 | 0.5129 | Yes |
| 136 | NEK2 |  |  | 194 | 0.423 | 0.5162 | Yes |
| 137 | MIS18A |  |  | 196 | 0.421 | 0.5175 | Yes |
| 138 | SYNE2 |  |  | 203 | 0.413 | 0.5084 | No |
| 139 | CCP110 |  |  | 204 | 0.411 | 0.5117 | No |
| 140 | ZWINT |  |  | 212 | 0.407 | 0.5004 | No |
| 141 | H2AFZ |  |  | 216 | 0.405 | 0.4974 | No |
| 142 | CENPO |  |  | 223 | 0.398 | 0.4882 | No |
| 143 | TIMELESS |  |  | 228 | 0.395 | 0.4830 | No |
| 144 | STIL |  |  | 231 | 0.393 | 0.4820 | No |
| 145 | POC1A |  |  | 234 | 0.390 | 0.4810 | No |
| 146 | EZH2 |  |  | 236 | 0.388 | 0.4820 | No |
| 147 | VRK1 |  |  | 238 | 0.388 | 0.4830 | No |
| 148 | PARPBP |  |  | 240 | 0.387 | 0.4840 | No |
| 149 | PCNA |  |  | 242 | 0.386 | 0.4850 | No |
| 150 | HMGN2 |  |  | 243 | 0.386 | 0.4881 | No |
| 151 | SUGP2 |  |  | 244 | 0.385 | 0.4912 | No |
| 152 | RTKN2 |  |  | 248 | 0.379 | 0.4880 | No |
| 153 | CCNB2 |  |  | 250 | 0.379 | 0.4889 | No |
| 154 | JADE1 |  |  | 251 | 0.378 | 0.4919 | No |
| 155 | MPHOSPH9 |  |  | 254 | 0.375 | 0.4908 | No |
| 156 | PLK4 |  |  | 255 | 0.374 | 0.4938 | No |
| 157 | RAD18 |  |  | 256 | 0.373 | 0.4967 | No |
| 158 | C18orf54 |  |  | 259 | 0.371 | 0.4956 | No |
| 159 | RFC5 |  |  | 260 | 0.370 | 0.4985 | No |
| 160 | CENPJ |  |  | 261 | 0.370 | 0.5014 | No |
| 161 | GAS2L3 |  |  | 262 | 0.369 | 0.5044 | No |
| 162 | TUBB |  |  | 263 | 0.368 | 0.5073 | No |
| 163 | CENPW |  |  | 267 | 0.366 | 0.5040 | No |
| 164 | TUBG1 |  |  | 268 | 0.365 | 0.5069 | No |
| 165 | CEP57L1 |  |  | 269 | 0.364 | 0.5098 | No |
| 166 | TUBA1C |  |  | 270 | 0.362 | 0.5127 | No |
| 167 | CHAF1A |  |  | 276 | 0.359 | 0.5052 | No |
| 168 | MMS22L |  |  | 277 | 0.359 | 0.5081 | No |
| 169 | ANP32E |  |  | 280 | 0.358 | 0.5068 | No |
| 170 | PSIP1 |  |  | 284 | 0.357 | 0.5034 | No |
| 171 | CDC25B |  |  | 286 | 0.356 | 0.5042 | No |
| 172 | CALM2 |  |  | 290 | 0.354 | 0.5008 | No |
| 173 | HAUS8 |  |  | 293 | 0.353 | 0.4995 | No |
| 174 | RFC4 |  |  | 295 | 0.351 | 0.5002 | No |
| 175 | SUZ12 |  |  | 297 | 0.349 | 0.5009 | No |
| 176 | NEIL3 |  |  | 304 | 0.345 | 0.4912 | No |
| 177 | DTL |  |  | 305 | 0.345 | 0.4940 | No |
| 178 | CDC25C |  |  | 307 | 0.343 | 0.4946 | No |
| 179 | CENPN |  |  | 309 | 0.343 | 0.4953 | No |
| 180 | PKP4 |  |  | 311 | 0.342 | 0.4960 | No |
| 181 | RANGAP1 |  |  | 312 | 0.342 | 0.4987 | No |
| 182 | DEK |  |  | 316 | 0.341 | 0.4952 | No |
| 183 | PHF19 |  |  | 319 | 0.339 | 0.4937 | No |
| 184 | RFC3 |  |  | 322 | 0.338 | 0.4923 | No |
| 185 | HAUS6 |  |  | 324 | 0.337 | 0.4929 | No |
| 186 | CIT |  |  | 326 | 0.336 | 0.4935 | No |
| 187 | HIRIP3 |  |  | 336 | 0.331 | 0.4775 | No |
| 188 | BTG3 |  |  | 337 | 0.331 | 0.4801 | No |
| 189 | PHGDH |  |  | 344 | 0.328 | 0.4703 | No |
| 190 | TMEM237 |  |  | 353 | 0.324 | 0.4564 | No |
| 191 | ARHGAP11B |  |  | 356 | 0.323 | 0.4548 | No |
| 192 | CCDC14 |  |  | 357 | 0.322 | 0.4574 | No |
| 193 | TACC1 |  |  | 359 | 0.322 | 0.4578 | No |
| 194 | FSTL1 |  |  | 362 | 0.321 | 0.4563 | No |
| 195 | LMNB1 |  |  | 363 | 0.321 | 0.4588 | No |
| 196 | PNRC2 |  |  | 364 | 0.321 | 0.4614 | No |
| 197 | PARP2 |  |  | 372 | 0.319 | 0.4494 | No |
| 198 | EFNB2 |  |  | 374 | 0.317 | 0.4499 | No |
| 199 | MID1 |  |  | 375 | 0.317 | 0.4524 | No |
| 200 | SOGA1 |  |  | 378 | 0.314 | 0.4508 | No |
| 201 | CEP128 |  |  | 381 | 0.314 | 0.4491 | No |
| 202 | ZNF714 |  |  | 382 | 0.313 | 0.4516 | No |
| 203 | PRIM2 |  |  | 391 | 0.311 | 0.4375 | No |
| 204 | KIF20A |  |  | 392 | 0.311 | 0.4400 | No |
| 205 | CEP192 |  |  | 395 | 0.310 | 0.4383 | No |
| 206 | EZR |  |  | 403 | 0.307 | 0.4263 | No |
| 207 | RHEB |  |  | 404 | 0.307 | 0.4287 | No |
| 208 | BLM |  |  | 408 | 0.305 | 0.4249 | No |
| 209 | CENPC |  |  | 409 | 0.304 | 0.4274 | No |
| 210 | PTTG1 |  |  | 413 | 0.302 | 0.4236 | No |
| 211 | ZGRF1 |  |  | 415 | 0.302 | 0.4239 | No |
| 212 | CDKN1B |  |  | 427 | 0.298 | 0.4035 | No |
| 213 | ZMYM1 |  |  | 429 | 0.298 | 0.4038 | No |
| 214 | NUP107 |  |  | 430 | 0.298 | 0.4062 | No |
| 215 | SASS6 |  |  | 432 | 0.296 | 0.4064 | No |
| 216 | PHIP |  |  | 440 | 0.295 | 0.3943 | No |
| 217 | G2E3 |  |  | 446 | 0.294 | 0.3863 | No |
| 218 | IQGAP3 |  |  | 447 | 0.294 | 0.3886 | No |
| 219 | CCDC150 |  |  | 449 | 0.293 | 0.3889 | No |
| 220 | MZT1 |  |  | 456 | 0.291 | 0.3788 | No |
| 221 | CBX5 |  |  | 457 | 0.291 | 0.3811 | No |
| 222 | SAE1 |  |  | 463 | 0.289 | 0.3731 | No |
| 223 | RFWD3 |  |  | 464 | 0.289 | 0.3754 | No |
| 224 | NUP50 |  |  | 470 | 0.287 | 0.3673 | No |
| 225 | KCTD9 |  |  | 471 | 0.287 | 0.3696 | No |
| 226 | CHEK1 |  |  | 477 | 0.286 | 0.3615 | No |
| 227 | RNASEH2A |  |  | 478 | 0.285 | 0.3638 | No |
| 228 | NUCKS1 |  |  | 483 | 0.285 | 0.3578 | No |
| 229 | APOLD1 |  |  | 487 | 0.284 | 0.3538 | No |
| 230 | POLH |  |  | 492 | 0.282 | 0.3478 | No |
| 231 | CTDSPL2 |  |  | 495 | 0.282 | 0.3459 | No |
| 232 | DBF4B |  |  | 498 | 0.281 | 0.3440 | No |
| 233 | PSMC3 |  |  | 506 | 0.280 | 0.3317 | No |
| 234 | FAM83D |  |  | 511 | 0.279 | 0.3257 | No |
| 235 | TTF2 |  |  | 514 | 0.278 | 0.3237 | No |
| 236 | EML1 |  |  | 515 | 0.278 | 0.3259 | No |
| 237 | CDC27 |  |  | 520 | 0.278 | 0.3199 | No |
| 238 | NFIC |  |  | 531 | 0.276 | 0.3014 | No |
| 239 | POLR2A |  |  | 545 | 0.273 | 0.2766 | No |
| 240 | SAP30 |  |  | 547 | 0.273 | 0.2767 | No |
| 241 | SCLT1 |  |  | 548 | 0.273 | 0.2789 | No |
| 242 | CALU |  |  | 552 | 0.272 | 0.2749 | No |
| 243 | NIF3L1 |  |  | 561 | 0.270 | 0.2604 | No |
| 244 | BRD8 |  |  | 562 | 0.270 | 0.2626 | No |
| 245 | CNTRL |  |  | 575 | 0.269 | 0.2399 | No |
| 246 | SAPCD2 |  |  | 578 | 0.268 | 0.2379 | No |
| 247 | ZNF682 |  |  | 585 | 0.267 | 0.2276 | No |
| 248 | CCDC18 |  |  | 586 | 0.267 | 0.2297 | No |
| 249 | ZNF850 |  |  | 589 | 0.266 | 0.2277 | No |
| 250 | ARHGEF39 |  |  | 595 | 0.264 | 0.2194 | No |
| 251 | CWF19L2 |  |  | 608 | 0.262 | 0.1967 | No |
| 252 | TBC1D31 |  |  | 616 | 0.261 | 0.1843 | No |
| 253 | PAX6 |  |  | 617 | 0.261 | 0.1863 | No |
| 254 | NDE1 |  |  | 623 | 0.259 | 0.1781 | No |
| 255 | CENPI |  |  | 630 | 0.258 | 0.1677 | No |
| 256 | ZNF85 |  |  | 635 | 0.258 | 0.1615 | No |
| 257 | HNRNPUL1 |  |  | 637 | 0.257 | 0.1614 | No |
| 258 | HEG1 |  |  | 639 | 0.257 | 0.1614 | No |
| 259 | EXOSC8 |  |  | 647 | 0.256 | 0.1490 | No |
| 260 | UHRF1 |  |  | 652 | 0.255 | 0.1427 | No |
| 261 | DESI2 |  |  | 654 | 0.255 | 0.1427 | No |
| 262 | NUP37 |  |  | 655 | 0.255 | 0.1447 | No |
| 263 | USP13 |  |  | 673 | 0.252 | 0.1115 | No |
| 264 | SPATA5 |  |  | 675 | 0.252 | 0.1114 | No |
| 265 | MYBL1 |  |  | 678 | 0.251 | 0.1093 | No |
| 266 | SGCE |  |  | 680 | 0.250 | 0.1092 | No |
| 267 | HMGCR |  |  | 683 | -0.257 | 0.1071 | No |
| 268 | CHD7 |  |  | 706 | -0.284 | 0.0638 | No |
| 269 | ID4 |  |  | 708 | -0.285 | 0.0640 | No |
| 270 | MYO10 |  |  | 710 | -0.305 | 0.0644 | No |
| 271 | ZEB1 |  |  | 717 | -0.317 | 0.0545 | No |
| 272 | PRDX1 |  |  | 719 | -0.326 | 0.0550 | No |
| 273 | PTMS |  |  | 720 | -0.333 | 0.0577 | No |
| 274 | TMEM256 |  |  | 724 | -0.345 | 0.0542 | No |
| 275 | ATCAY |  |  | 738 | -0.417 | 0.0306 | No |
| 276 | HES6 |  |  | 752 | -0.523 | 0.0079 | No |
| 277 | DANCR |  |  | 753 | -0.572 | 0.0124 | No |
Table: GSEA details [plain text format]

  

Fig 2: RICHARDS\_NOWAKOWSKI\_SCIENCE\_2017\_IPC\_DIV1\_UPREG: Random ES distribution      
 Gene set null distribution of ES for **RICHARDS\_NOWAKOWSKI\_SCIENCE\_2017\_IPC\_DIV1\_UPREG**
